# Strategies to rare primary cardiac lipomas in the left ventricle in a patient: case report

**DOI:** 10.1186/s12872-022-02748-w

**Published:** 2022-07-15

**Authors:** Wei Liu, Haisong Bu

**Affiliations:** Department of Cardiovascular Surgery, Xiangya Hospital, Central South University, 87 Xiangya Road, Changsha, 410008 Hunan People’s Republic of China

**Keywords:** Cardiac tumors, Benign, Left ventricle, Image, Surgery

## Abstract

**Background:**

Primary cardiac tumors are rare in all age groups and are usually benign. Symptoms are usually related to tumor size, location, invasiveness, number, and growth rate. While histologically benign, cardiac arrest may be caused by blocked inflow or outflow or malignant ventricular arrhythmia. Surgical resection of left ventricular tumors, especially those involving the outflow tract, is challenging.

**Case presentation:**

Herein, we present a rare case of an asymptomatic, 39-year-old woman who was referred to our cardiovascular department for a huge left ventricular cardiac mass incidentally discovered during the physical examination. Images showed a huge mass that quasi-circular low-density focus with a clear boundary and regular shape in the left ventricular cavity and fortunately had no significant effect on the peripheral valves and hemodynamics.

**Conclusions:**

This illustrative report highlights the exact surgical management of a cardiac tumor depends largely on the site and extent of the mass. Mechanical compromise and not the neoplastic potential should be considered. A conservative approach and follow-up regularly are advocated to ensure that the patient gets the best diagnosis and treatment, however, surgery is indicated only for severely symptomatic patients with hemodynamic compromise.

## Background

Primary cardiac tumors are rare in all age groups and are usually benign [1]. Symptoms are usually related to tumor size, location, invasiveness, number, and growth rate [2, 3]. The general symptoms were tumor-related intracardiac obstruction, compression of extracardiac large vessels, distal embolism of tumor fragments or adherent thrombi, and local tumor infiltration [4]. Cardiac tumors are usually difficult to find in the early stage and have no obvious symptoms. Most of them are found in routine screening or physical examination. While histologically benign, cardiac arrest may be caused by blocked inflow or outflow or malignant ventricular arrhythmia [3]. The surgical treatment strategy of cardiac tumor mainly depends on the patient's symptoms and the location of the mass. Surgical resection of left ventricular tumors, especially those involving the outflow tract, is challenging [5]. However, partial resection and asymptomatic conservative treatment strategies have been paid more and more attention [3].

## Case presentation

**Fig. 1:**
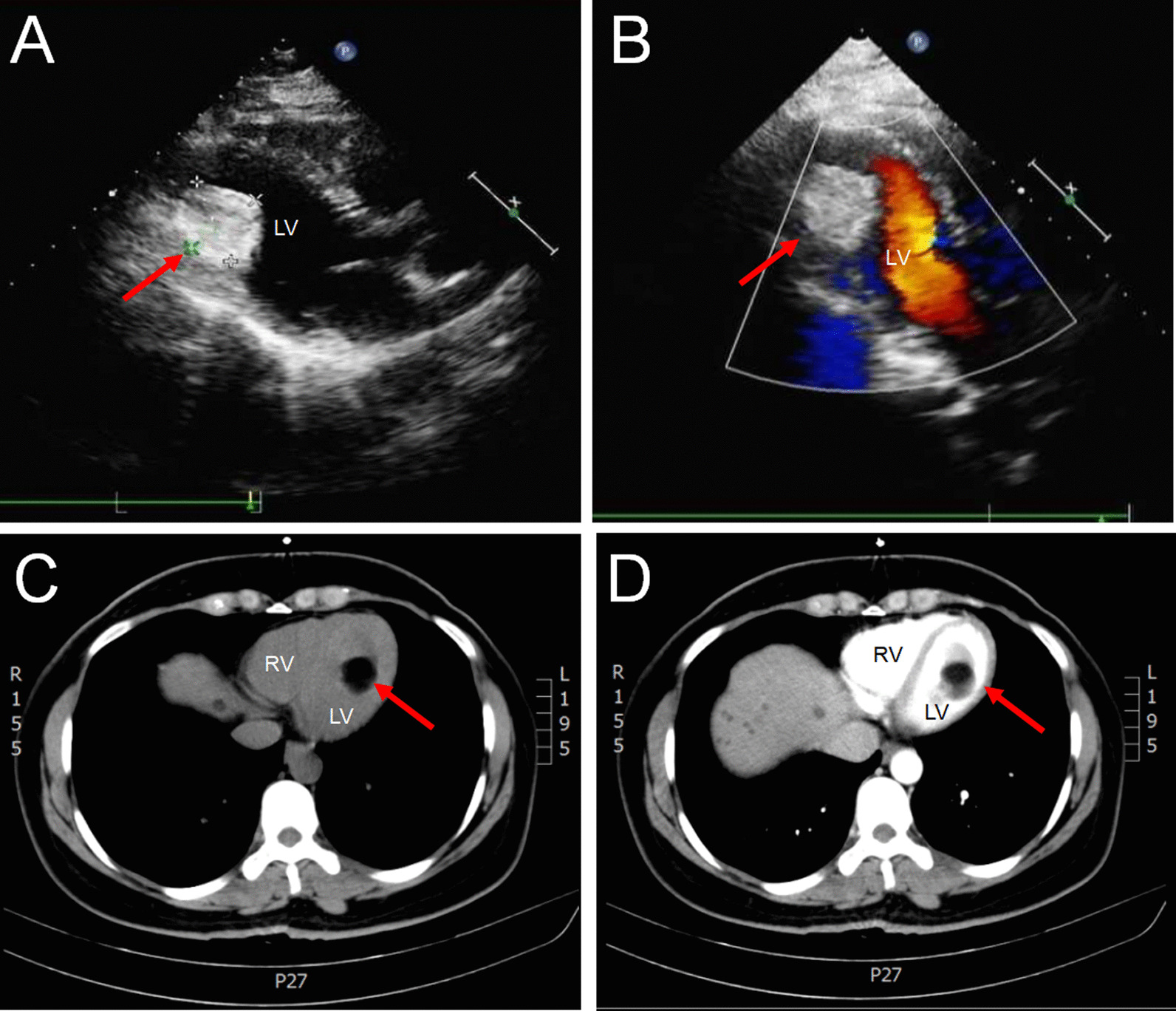
Echocardiography revealed an echo-bright mass in the LV (**A**). Color-flow Doppler echocardiography showed no significant effect on the peripheral valves and hemodynamics (**B**). Cardiac computed tomography was performed and revealed a quasi-circular low-density focus with a clear boundary and regular shape in the LV (**C** and **D**). LV: left ventricle; RV: right ventricle

**Fig. 2:**
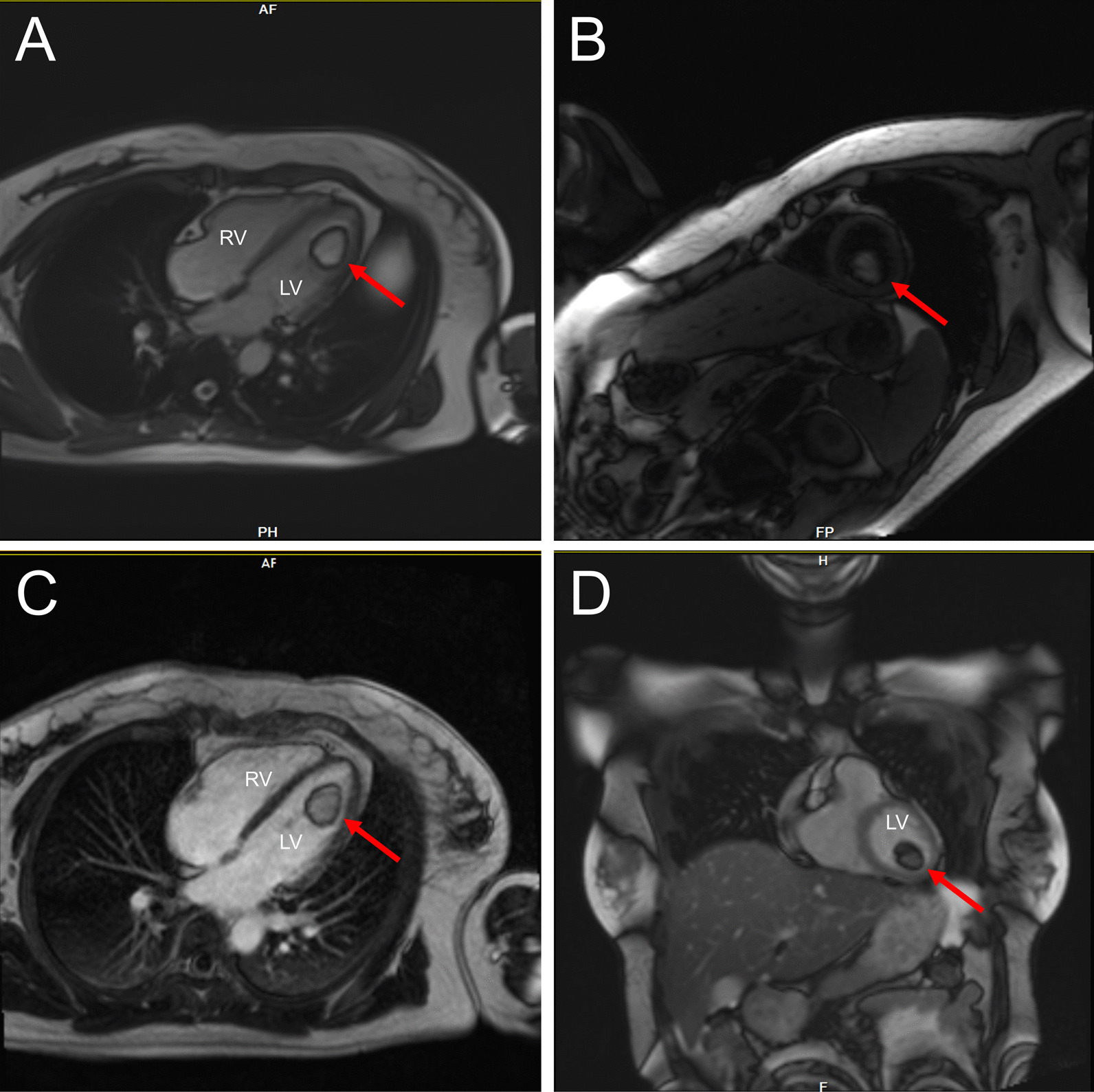
Magnetic resonance imaging confirmed a huge low signal non-enhancing lesions in the LV with a clear boundary and no obvious enhancement after perfusion (**A**–**D**). LV: left ventricle; RV: right ventricle

An asymptomatic, 39-year-old woman was referred to our cardiovascular department for a huge left ventricular cardiac tumor incidentally discovered during the physical examination. Occasional shortness of breath after activity was dictated. Physical examination revealed that the heart rate was 76 bpm with breathing 23 times/min. There were no other meaningful clinical manifestations. Echocardiography showed a huge mass in the left ventricle (approximately 30 mm × 35 mm) (Fig. 1A and B, arrows) that had no significant effect on the peripheral valves and hemodynamics. The diameters of the right ventricle (RV), the left ventricle (LV), and left ventricular posterior wall (LVPW) were 15, 48, and 9, respectively. The left ventricular systolic function was normal with an ejection fraction of 62%. Cardiac computed tomography (CT) was performed and revealed a quasi-circular low-density focus with a clear boundary and regular shape in the left ventricular cavity (Fig. 1C and D, arrows). Cardiac magnetic resonance imaging (MRI) was also confirmed a no obvious enhancement lesion after perfusion in the LV with a clear boundary and exhibited a lipoma characteristic appearance (Fig. 2A–D, arrows). Based on the clinical symptoms and imaging results, our initial diagnosis was benign cardiac tumor: lipoma? Following communication with the patient, the patient refused surgical treatment and would perform conservative treatment strategies due to the lack of clinical symptoms support, the characteristics of lipoma disease and coupled with the heavy economic burden. The patient was followed up regularly. A MRI Brain should also be done in follow up to assess any occult embolization to cerebral microcirculation.

## Discussion and conclusions

Primary cardiac tumors are rare disease, and 80% of it is histologically benign [6]. According to the autopsy series, the incidence fluctuated between 0.0017 and 0.28% [7]. Symptoms are usually related to tumor size, location, invasiveness, number, and growth rate [3]. If symptoms occur, they are usually nonspecific, such as heart murmur, arrhythmia, dyspnea, and congestive heart failure [8].

Echocardiography is the primary imaging technique for the evaluation of cardiac tumors. Tumor location, extent, and characteristics (single or multiple, intramuscular or intracavitary, solid or cystic) can be evaluated accurately and rapidly. Color-flow Doppler echocardiography is of great significance in evaluating the obstructive nature and hemodynamics of cardiac tumors [9]. Echocardiography has a strong sensitivity to intraluminal tumors, while other types may need to be combined with other detection methods [10]. Cardiac CT or MRI provides complementary information to echocardiography. MRI helps to elucidate the relationship of the tumor to the normal myocardium and the great vessels [11]. It can not only provide information about tumor location, size and boundary, but also identify some tumor tissue types, such as lipoma, to enhance its value as a non-invasive diagnostic tool [12]. This feature is particularly valuable in planning the extent and feasibility of tumor resection.

Although the surgical treatment strategy of cardiac tumor mainly depends on the patient's symptoms and the location of the mass, some general comments regarding surgical resection are appropriate. Surgical resection of left ventricular tumors, especially those involving the outflow tract, is challenging [5]. In the interest of avoiding a left ventriculotomy, some tumors may be excised via a retrograde technique involving an aortotomy and retraction of the aortic valve leaflets. Ross operation is mainly applicable to the left ventricular outflow tract and aortic valve involvement [13]. Benign cardiac tumors are non-invasive, so we should focus on mechanical injury and reduce the concern of tumor potential [14]. Rhabdomyomas are the most common tumor type, and most patients have spontaneous regression [15].Therefore, a conservative approach is advocated to allow the tumor to regress, and surgery is indicated only for severely symptomatic patients with hemodynamic compromise or patients with refractory dysrhythmias [16].

For heart tumors, surgeons usually try to remove them completely. Some doctors even indicate that the primary surgical goal should be to remove the tumor as radically as possible and remove any potential obstacles in any case [17]. Extensive myocardial involvement of the tumor or its proximity to critical structures (e.g., coronary arteries or valves) may preclude complete excision. However, partial resection can also achieve satisfactory surgical results [3]. Thus, total resection of the tumor is not the only therapeutic aim, and it is more important to maintain good cardiac function, such as partial resection and conservative approach [14].

This illustrative report highlights cardiac lipomas are rare and histologically benign, and they have a high propensity to cause obstruction, arrhythmias or cardiac arrest. However, the exact surgical management of a cardiac tumor depends largely on the site and extent of the mass. Mechanical compromise and not the neoplastic potential should be considered. A conservative approach and follow-up regularly are advocated to ensure that the patient gets the best diagnosis and treatment, however, surgery is indicated only for severely symptomatic patients with hemodynamic compromise.

## Data Availability

The datasets used and/or analyzed during the current study are available from the corresponding author on reasonable request.

## Abbreviations

RV: Right ventricle
LV: Left ventricle
LVPW: Left ventricular posterior wall
CT: Computed tomography
MRI: Magnetic resonance imaging
